# Prostate-specific antigen and hormone receptor expression in male and female breast carcinoma

**DOI:** 10.1186/1746-1596-5-63

**Published:** 2010-09-23

**Authors:** Teresa S Kraus, Cynthia Cohen, Momin T Siddiqui

**Affiliations:** 1 Department of Pathology and Laboratory Medicine, Emory University, Atlanta, GA, USA

## Abstract

**Background:**

Prostate carcinoma is among the most common solid tumors to secondarily involve the male breast. Prostate specific antigen (PSA) and prostate-specific acid phosphatase (PSAP) are expressed in benign and malignant prostatic tissue, and immunohistochemical staining for these markers is often used to confirm the prostatic origin of metastatic carcinoma. PSA expression has been reported in male and female breast carcinoma and in gynecomastia, raising concerns about the utility of PSA for differentiating prostate carcinoma metastasis to the male breast from primary breast carcinoma. This study examined the frequency of PSA, PSAP, and hormone receptor expression in male breast carcinoma (MBC), female breast carcinoma (FBC), and gynecomastia.

**Methods:**

Immunohistochemical staining for PSA, PSAP, AR, ER, and PR was performed on tissue microarrays representing six cases of gynecomastia, thirty MBC, and fifty-six FBC.

**Results:**

PSA was positive in two of fifty-six FBC (3.7%), focally positive in one of thirty MBC (3.3%), and negative in the five examined cases of gynecomastia. PSAP expression was absent in MBC, FBC, and gynecomastia. Hormone receptor expression was similar in males and females (AR 74.1% in MBC vs. 67.9% in FBC, p = 0.62; ER 85.2% vs. 68.5%, p = 0.18; and PR 51.9% vs. 48.2%, p = 0.82).

**Conclusions:**

PSA and PSAP are useful markers to distinguish primary breast carcinoma from prostate carcinoma metastatic to the male breast. Although PSA expression appeared to correlate with hormone receptor expression, the incidence of PSA expression in our population was too low to draw significant conclusions about an association between PSA expression and hormone receptor status in breast lesions.

## Introduction

Primary carcinoma of the male breast accounts for less than 1% of cancers in men[1,2]. Uncommonly, metastatic malignancies may also involve the male breast[1,3].

Extramammary malignancies metastatic to the breast reportedly represent 0.46 - 5% of all malignant breast fine needle aspiration biopsies in females and males[4]. Incidence rates of metastasis to the breast as high as 6.6% have been reported when hematologic malignancies are included[5]. Prostatic carcinoma is among the most commonly reported solid tumors to secondarily involve the male breast[2]. In one study, 26% of patients with a known history of prostate carcinoma were found to have metastases to the breast at autopsy[6].

Metastatic neoplasms to the breast generally occur in the setting of disseminated metastatic disease, however patients may present with a breast mass as the first manifestation of metastatic cancer[4]. Metastatic malignancies involving the breast are associated with a poor prognosis, with the majority of patients dying within one year of diagnosis[7-9]. Treatment generally involves systemic chemotherapy, and correct diagnosis is essential to provide accurate prognostic information and prevent unnecessary surgery in these patients.

Reports of prostate specific antigen (PSA) expression in male and female breast carcinomas[3,10-15] have raised questions about the value of PSA staining in differentiating metastatic prostatic carcinoma from primary breast carcinoma. PSA expression has been reported to correlate with expression of androgen receptor (AR), estrogen receptor (ER) and progesterone receptor (PR) in some studies[10,13], however other studies have found no correlation[12].

The aim of this study was to assess the frequency of PSA and prostate specific acid phosphatase (PSAP) expression in male breast carcinoma (MBC), female breast carcinoma (FBC) and gynecomastia and to correlate these findings with hormone receptor expression.

## Methods

A search of electronic medical records from the Emory University Healthcare system was conducted to identify cases of MBC, FBC, and gynecomastia. With Institutional Review Board approval from Emory University (IRB00012568, 517-2002), tissue microarrays (TMAs) representing six cases of gynecomastia (four 0.1-cm cores per case), thirty MBC (four cores per case), and fifty-six FBC (two cores per case) were constructed from paraffin-embedded tissue blocks of surgical specimens.

### Immunohistochemistry

Immunohistochemical staining for PSA, PSAP and AR, ER, and PR were performed on 5-micron sections from TMA blocks. Antibody clones and dilutions are listed in Table 1. Sections are tested for the presence of primary antibody using the Dako Envision+dual link system which is an horseradish peroxidase labeled polymer (Dako, Carpinteria, CA) with heat-induced antigen retrieval.

**Table 1:** Antibodies and Interpretation Criteria

Antibody	Source	Clone	Dilution	Positive Control	Positive Result
PSA	Dako	Polyclonal	1:10,000	Prostate	Any cytoplasmic staining

PSAP	Dako	PASE/4LJ	1:1,280	Prostate	Any cytoplasmic staining

AR	Dako	F39.4.1	1:40	Prostate	Nuclear staining ≥ 10%

ER	Dako	ID5	1:50	Breast carcinoma	Nuclear staining ≥ 10%

PR	Dako	PgR636	1:400	Breast carcinoma	Nuclear staining ≥ 10%

PSA - prostate specific antigen; PSAP - prostate specific acid phosphatase; AR - androgen receptor; ER - estrogen receptor; PR - progesterone receptor.

The sections were deparaffinized and rehydrated in deionized water. Antigen retrieval was in heated citrate buffer (pH 6.0), using an electric pressure cooker for 3 minutes at 12-15 pounds per square inch (approximately 120°C); slides were cooled for 10 minutes prior to immunostaining.

The slides were loaded on the Dako AutoStainer Plus (Dako) and exposed to 3% hydrogen peroxide for five minutes, incubated at room temperature with primary antibody for 30 minutes, followed by labeled polymer (Envision+dual link) for thirty minutes, 3,3'-diaminobenzidine as a chromogen for five minutes, and hematoxylin counterstain for five minutes.

### Interpretation

PSA and PSAP were considered positive if any cytoplasmic staining was observed. Positive reactions for AR, ER, and PR were defined by nuclear staining observed in at least 10% of tumor cells.

### Statistical analysis

P-values for hormone receptor status of FBC and MBC were determined by Fisher's exact test.

## Results

Immunohistochemical staining results are summarized in Table 2. PSA was positive in two of 56 FBC (3.7%), and focally positive (< 1% of cells) in one of 30 MBC (3.3%). Hormone receptor expression was similar in males and females (p = 0.62, 0.18, and 0.82 for AR, ER, and PR, respectively).

**Table 2:** PSA, PSAP, and Hormone Receptor Expression in MBC, FBC, and Gynecomastia.

Marker	Gynecomastia	MBC	FBC	P-value (MBC vs FBC)
PSA	0/5 (0%)	1/30 (3.3%)	2/54 (3.7%)	1.00

PSAP	0/5 (0%)	0/29 (0%)	0/54 (0%)	1.00

AR	4/5 (80%)	20/27 (74.1%)	38/56 (67.9%)	0.62

ER	6/6 (100%)	23/27 (85.2%)	37/54 (68.5%)	0.18

PR	5/5 (100%)	14/27 (51.9%)	27/56 (48.2%)	0.82

MBC - male breast carcinoma; FBC - female breast carcinoma; PSA - prostate specific antigen; PSAP - prostate specific acid phosphatase; AR - androgen receptor; ER - estrogen receptor; PR - progesterone receptor.

**Figure 1:**
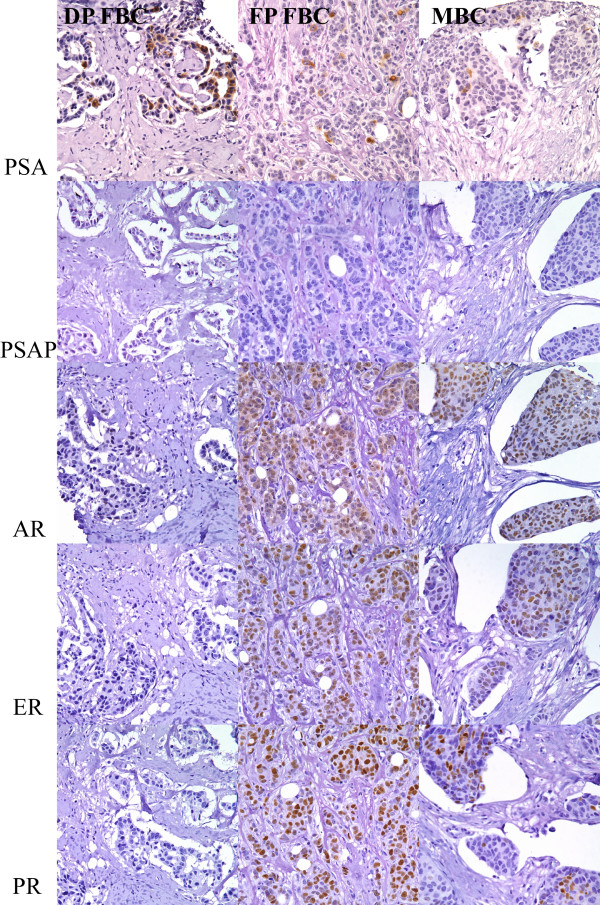
**Results of immunohistochemical stains in PSA-positive breast carcinomas**. DP FBC - Diffusely PSA-positive female breast carcinoma; FP FBC - Focally PSA-positive female breast carcinoma; MBC - PSA-positive male breast carcinoma; PSA - prostate specific antigen; PSAP - prostate specific acid phosphatase; AR - androgen receptor; ER - estrogen receptor; PR - progesterone receptor.

Of the two PSA-positive FBC cases, one was positive for AR, ER, and PR, and the other was negative for AR (fewer than 10% of nuclei staining), ER and PR (Figure 1). The PSA-positive MBC was positive for AR, ER, and PR. The number of PSA-positive cases was too small to draw any significant conclusions about the correlation between PSA expression and hormone receptor status.

The clinicopathologic features of the PSA-positive cases are listed in Table 3. One of the three PSA-positive cases was well-differentiated (histologic grade I), and the remaining cases were poorly differentiated (histologic grade III).

**Table 3:** Grading and Staging Data for PSA-Positive Cases

Age/Sex	Nuclear Grade	Tumor Size	Lymph Node Status	Immunohistochemistry
62 F	I	1.5 cm	Negative (0/18)	PSA+ (diffuse), PSAP-, AR-, ER-, PR-

75 F	III	2.5 cm	Negative (0/11)	PSA+ (focal), PSAP- AR+, ER+, PR+

58 M	III	1.3 cm	Positive (2/13)	PSA+ (focal), PSAP-, AR+, ER+, PR+

PSA - prostate specific antigen; PSAP - prostate specific acid phosphatase; AR - androgen receptor; ER - estrogen receptor; PR - progesterone receptor.

## Discussion

Prostate carcinoma has been reported to be the most common metastatic malignancy to the male breast[2]. Lung cancer and melanoma are also among the commonly reported neoplasms that secondarily involve the male breast[16]. While the treatment for primary breast carcinoma involves surgical resection, treatment of metastatic carcinoma to the breast generally involves systemic therapy. Thus, accurate diagnosis is essential to prevent unnecessary surgery and provide optimal treatment and prognostic information to these patients.

Radiographic clues may aid in the differentiation of primary breast carcinoma from metastatic disease, as primary lesions tend to be spiculated and metastases are often more discrete and rounded[4,5,7,8]. Calcifications are more likely to occur in primary breast carcinomas[5,7,8]. Histologically, the presence of carcinoma in situ and elastosis are more commonly encountered in primary breast carcinoma than in metastatic tumors. Nonetheless, metastatic malignancies may closely mimic primary breast carcinoma in imaging studies and on histologic examination, and definitive diagnosis often depends on immunohistochemical studies[1].

PSA and PSAP are commonly used to suggest prostatic origin of malignant cells. PSA is a 33 kD glycoprotein with serine protease activity[11]. The detection of PSA in tissues by immunohistochemical staining is widely used to confirm the prostatic origin of carcinoma. Though initially thought to be specific to the prostate gland, PSA has been detected in other tissues, including the parotid, pancreas, and kidney, carcinomas of the ovary, lung, and breast[15], neuroendocrine cells[17], and benign breast lesions[11].

PSAP is a tyrosine phosphatase involved in growth regulation, and is regarded as a less sensitive, more specific marker of prostatic origin of cells[2]. According to Erbas *et al*., PSAP was detected in breast cyst fluid samples by ELISA;[18] however, immunohistochemically detectable PSAP expression was not identified in breast carcinomas or gynecomastia in previous studies[2,3,12,19].

Immunohistochemical detection of PSA and PSAP has been useful in the diagnosis of prostate carcinoma metastasis to the breast. Cheng *et al.*[3] reported one such case, and reviewed 33 additional cases from the literature. PSA expression was identified in 12 of 12 cases (100%) and PSAP expression in 13 of 14 cases (92.8%) where immunohistochemistry was performed.

Our series represents the largest group of MBCs to be tested for immunohistochemically detectable PSA expression, performed in conjunction with a significant number of FBCs.

In our population, PSA expression was not detected in gynecomastia, and was uncommon in both male and female breast cancers. PSAP expression was not detected in benign or malignant breast tissue, similar to previous reports [2,3,12,19].

Table 4 summarizes the results of previous studies that examined PSA expression in breast lesions. The reported frequency of PSA-positive breast carcinoma varies from 0%[3,19,20] to 100%[21]. As similar antibodies were used in many of these studies, the reason for this discrepancy is not entirely clear, but may be related to differences in immunohistochemical staining protocols. Of note, Ilvan *et al.*[10] and Miller *et al.*[11] used monoclonal PSA antibodies in their studies, and reported similar low rates of PSA expression (10.1% and 9% of breast cancer cases, respectively).

**Table 4:** Previous Reports of Immunohistochemically Detectable PSA Expression in Benign and Malignant Breast Lesions

Author	Number of Cases	Antibody, Clone	PSA Positive	Comments
Carder *et al. *2005 [2]	11 MBC	Dako, ELY, Cambridgeshire, UK	1/11 (9%)	Focal intense staining All cases PSAP negative (Dako)

Cheng *et al. *2006 [3]	14 MBC	Dako, Glostrup, Denmark	0/14	All cases PSAP negative

Ilvan *et al. *2004 [10]	109 FBC	Dako USA, monoclonal, prediluted	11/109 (10.1%)	All PSA positive cases ER+ PR+

Miller *et al. *2001 [11]	75 (74 FBC, 1 MBC)	Dako M0750 1:320	7/75 (9%)	5/7 showed weak, focal positivity; MBC negative

Kidwai *et al. *2003 [12]	26 MBC	Dako polyclonal 1:1800	6/26 (23%)	5 focally positive 1 diffuse All PSAP negative

Narita *et al. *2006 [13]	156 FBC	Dako polyclonal, ready to use	61/156 (39.1%)	Positive correlation with AR expression

Hall *et al. *1998 [14]	72 cases	Dako, Carpinteria, CA	44/72 (61%)	

Alanen *et al. *1999 [15]	171 cases	Dakopatts, Glostrup, Denmark, polyclonal 1:1000	54/171 (31.6%)	

Gatalica *et al. *1999 [19]	18 gynecomastia, 8 MBC	Rabbit polyclonal, Ventana Medical Systems, Tucson AZ	5/18 cases of gynecomastia (27.8%) 0/8 MBC	Focal strong polyclonal PSA; 4/5 cases tested with monoclonal PSA and were negative All cases PSAP (Ventana PASE/4LT) negative

Quraishi *et al. *2007 [20]	30 (8 DCIS, 19 infiltrating ductal carcinoma, 3 metastatic breast carcinoma)	Polyclonal rabbit	0/30	

Gupta 1999 [21]	2 MBC	Not reported	2/2 (100%)	Cell blocks positive; trace PSA positivity in resection specimens

Tanaka *et al. *2002 [22]	78 FBC	Dako A/S Glostrup, Denmark, polyclonal 1:100	29/78 (32.7%)	6 cases strongly positive

This article	54 FBC 30 MBC 5 gynecomastia	Dako polyclonal 1:10,000	2/54 FBC 1/30 MBC 0/5 gynecomastia	All cases PSAP negative

MBC - male breast carcinoma; FBC - female breast carcinoma; PSA - prostate specific antigen; PSAP - prostate specific acid phosphatase; AR - androgen receptor; ER - estrogen receptor; PR - progesterone receptor.

The frequency of PSA expression in our series of breast carcinomas was lower than in a report by Kidwai *et al. *(2003), where PSA positivity was observed in six of 26 cases of MBC (23%)[12]. Although staining for PSA was focal in five of the positive cases, one case was diffusely positive. The polyclonal PSA antibody used in our study was similar to that used by Kidwai *et al. *Other studies, most of which focused predominantly on FBC, have reported results similar to those described by Kidwai *et al.*[3,10,13,15,22]

Our results are similar to those reported by Carder *et al.*[2] and Gatalica *et al*.,[19] who noted PSA positivity in one of eleven MBCs (9.1%) and zero of eight MBCs (0%), respectively. In the latter study, five of eighteen cases of gynecomastia (27.8%) showed staining for polyclonal PSA. When a monoclonal PSA antibody was used, however, only one case remained positive (5.6%)[19].

Based on previous reports, PSA expression appears to be less common in MBC than in FBC. When taken together, the overall incidence of PSA expression in breast cancer cases reported in the literature is 28.5% (215 of 754 total cases; range 0%[3,19,20] to 100%)[21]. When only MBC is considered, the incidence is 14.5% (9 of 62 cases; range 0%[3,19] to 100%)[21]. When our cases are included, the overall incidence is 26% (218 of 836 cases) and the incidence in MBC is 11% (10 of 92 cases). In contrast to previous studies, the percentage of PSA-positive FBC cases was similar to the percentage of PSA-positive MBC cases in our study.

The physiologic significance of PSA expression in extraprostatic tissues is not known. Though PSA has been shown to hydrolyze or otherwise modify substrates such as fibrinogen and laminin[23], its function (if any) in breast tissue and its prognostic significance in breast carcinoma remain unclear. Previous reports have suggested a relationship between PSA expression and carcinomas of low histologic grade[14,15]. In the current study, PSA expression did not appear to be associated with grade. PSA-positive tumors were either well-differentiated or poorly differentiated, and PSA-negative tumors were fairly evenly distributed between histologic grades I (23%), II (39%), and III (38%) (data not shown).

PSA positivity in breast cancer cells has also been reported to correlate with hormone receptor positive status[10] and possibly with improved prognosis[11], although other studies have found no such association[12]. PSA expression has been induced by androgens, progesterone, and glucocorticoids in steroid receptor-positive breast cancer cell lines[24], suggesting that PSA expression in breast tumors is indeed under hormonal control. In a study of 61 PSA-positive FBC, Narita *et al. *reported that 57 cases (93.4%) were AR-positive, 28 (46%) were ER-positive, and 22 (36%) were PR-positive[13]. In our study, two of three PSA-positive carcinomas were positive for AR, ER, and PR, and the hormone receptor-negative case demonstrated focal weak AR expression that did not meet our 10% threshold for AR positivity. Our results suggest a correlation between PSA expression and expression of hormone receptors, though the incidence of PSA expression in our population was too low to draw any significant conclusions about this association.

## Conclusions

Our data suggest that immunohistochemically detectable expression of PSA and PSAP in male and female breast carcinomas is infrequent. Thus, PSA and PSAP remain viable and useful markers for differentiating primary breast carcinoma from metastatic prostatic carcinoma involving the male breast. As with any immunohistochemical stain, however, these markers should be used in conjunction with clinical history, radiology, and histologic findings.

## Competing interests

The authors declare that they have no competing interests.

## Authors' contributions

MTS conceived of the study, and participated in its design and coordination. CC participated in the design of the study, case selection, tissue microarray construction and immunohistochemical staining. TSK reviewed the tissue microarray slides, performed data analysis, and drafted the manuscript. All authors read and approved the final manuscript.
